# In‐depth cardiovascular and pulmonary assessments in children with multisystem inflammatory syndrome after SARS‐CoV‐2 infection: A case series study

**DOI:** 10.14814/phy2.15201

**Published:** 2022-03-11

**Authors:** Camilla Astley, Maria Fernanda Badue Pereira, Marcos Santos Lima, Carlos Alberto Buchpiguel, Camila G. Carneiro, Marcelo Tatit Sapienza, Gabriela Nunes Leal, Danilo Marcelo Leite do Prado, Tiago Peçanha, Sofia Mendes Sieczkowska, Olivia Mari Matsuo, Livia Lindoso, Heloisa Helena Marques, Clovis Artur Silva, Bruno Gualano

**Affiliations:** ^1^ Applied Physiology and Nutrition Research Group School of Physical Education and Sport University of Sao Paulo Sao Paulo Brazil; ^2^ Rheumatology Division Clinical Hospital School of Medicine University of Sao Paulo Sao Paulo Brazil; ^3^ Chidren and Adolescent Institute Clinical Hospital School of Medicine University of Sao Paulo Sao Paulo Brazil; ^4^ Nuclear Medicine Division Department of Radiology and Oncology Clinical Hospital School of Medicine University of Sao Paulo Sao Paulo Brazil; ^5^ Food Research Center University of Sao Paulo Sao Paulo Brazil

**Keywords:** cardiovascular imaging, COVID‐19, inflammation, MIS‐C, children

## Abstract

We assessed PET‐CT myocardial blood flow (MBF) using N‐13 ammonia, brachial flow‐mediated dilation, and cardiopulmonary exercise test in five post‐discarged MIS‐C survivors. None of the patients (median age: 9, range: 7‐18 years; 3 females; 2 males) had preexisting pediatric chronic conditions. At the follow‐up visit, two patients exhibited severe perfusion defect developed in the left ventricular cavity, suggesting extensive myocardial ischemia (MBF <2.0) and one patient showed persistent mild pericardial effusion. Others two patients demonstrated endothelial dysfunction. Nevertheless, all patients had lower predicted values in the *V*O_2peak_, *V*O_2VAT_, OUES, and O_2_ Pulse (range: 35.2%–64.5%; 15.6%–38.2%; 1.0–1.3 L/min; 4–7 ml/beat), respectively. Our d suggested that previously health MIS‐C patients had impaired MBF, endothelial dysfunction and lower cardiopulmonary capacity at follow‐up analysis. Multidisciplinary further investigations should be conducted to reinforce these findings.

## Graphical abstract

Previously health multisystem inflammatory syndrome in children (MIS‐C) patients showed impaired myocardial flow reserve, endothelial dysfunction, and lower cardiopulmonary capacity after 1.9 months of infection. Our findings reveal novel pathophysiological findings in MIS‐C patients, which advances the knowledge on this newly described condition and may help tailor better treatments for these patients.

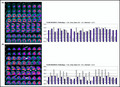

## 1 INTRODUCTION

Multisystem inflammatory syndrome in children (MIS‐C) is a hyperinflammatory response that commonly develops within 2–6 weeks following severe acute respiratory syndrome coronavirus 2 (SARS‐CoV‐2) infection, requiring hospitalization in the acute phase (Ballin & Nordström, 2021). While multiple cohorts have shown that MIS‐C patients may present with heterogeneous signs that include hemodynamic instability, tachycardia, left ventricular dysfunction, and respiratory distress, possibly primary or caused by cardiac dysfunction (Bateman et al., 2021; Bongers et al., 2011) the pathophysiology of MIS‐C and its natural course are yet to be fully elucidated.

Herein we report on a broad, in‐depth assessment of cardiovascular and pulmonary outcomes in a series of post discharged MIS‐C survivors, which unravels novel pathological features associated with this syndrome.

## 2 METHODS

### 2.1 Study design and patients

This case series is part of a prospective cohort study aimed at exploring the spectrum of the long‐term effects of COVID‐19 and MIS‐C in the pediatric population (clinicaltrials.gov NCT04659486). Data of the patients’ acute phase were retrospectively assessed through medical records. The post‐discharge data were collected prospectively in a dedicated, multidisciplinary, outpatient clinic for COVID‐19 (*n* = 130) and MIS‐C (*n* = 16) at the Children’ and Adolescents’ Institute of the Clinical Hospital of the University of Sao Paulo, between October 2020 and July 2021. Out of the 16 MIS‐C patients followed at our clinic, 4 died, 4 did not meet inclusion criteria (3 were younger than 7 years and 1 had type I diabetes mellitus), and 3 did not accept to participate. Therefore, five patients were included. All patients (median age: 9, range: 7–18 years; 3 females) fulfilled the MIS‐C diagnosis according to the Center for Disease Control (CDC) criteria (CDC, 2020). Four patients had positive serologic tests (ELISA assay to detect IgG for SARS‐CoV‐2), and one had a negative serologic test but was exposed to a confirmed COVID‐19 case within 4 weeks prior to the onset of symptoms. None of the patients had any preexisting pediatric chronic conditions. Patients’ main characteristics at hospital admission (during the acute phase) are shown in Table 1. Four out of five patients were admitted to the pediatric intensive care unit. Two patients required respiratory support and oxygen therapy, and three had vasodilatory shock. The median length stay was 12 (range: 3–18) days. The median time elapsed from discharge to the follow‐up visit was 1.9 (range: 1.3–6.2) months. At the follow‐up visit, we conducted a battery of assessments as follows: ^13^N‐ammonia PET‐CT imaging, standard echocardiography, brachial flow‐mediated dilation (FMD) using a Doppler ultrasound, maximal cardiopulmonary exercise test, and blood markers (C‐reactive protein, D‐dimer, fibrinogen, and troponin T). This study was approved by the local ethics committee (protocol #37460620.8.0000.0068) and registered at ClinicalTrials.gov (NCT04659486). Patients and guardians signed informed consent to participate in the study.

**TABLE 1:** Clinical features among MIS‐C patients

Patient's characteristics	Mean (SD), median (range) or *N* (%)	P1	P2	P3	P4	P5
Sex (female)	3 (60)	Female	Male	Female	Female	Male
Age (years)	10.2 (3.56)	16	7	9	8	11
Previously medical history	0 (0)	None	None	None	None	None
BMI (kg/m^2^)	20.1 (3.51)	24.7	21.2	18.3	21.1	15.3
Height (cm)	140 (0.11)	156	126	145	135	138
Weight (kg)	40.0 (11.9)	60.3	33.7	38.6	38.4	29.3
Signs and symptons at admission						
Fever (days)	7.60 (4.72)	Yes (12)	Yes (8)	Yes (12)	Yes (1)	Yes (5)
Conjutivitis	3 (60)	Yes	Yes	No	No	Yes
Hypotension	4 (80)	Yes	Yes	Yes	No	Yes
Shock	3 (60)	No	Yes	Yes	No	Yes
Abdominal pain	5 (100)	Yes	Yes	Yes	Yes	Yes
Diarrhea	3 (60)	Yes	No	No	Yes	Yes
Treatment						
ICU admission	4 (80)	No	Yes	Yes	Yes	Yes
Length of stay at hospital (days)	10.4 (6.26)	3	14	18	5	12
Respiratory support/oxygen therapy	2 (40)	No/No	Yes/Yes	No/No	No/No	Yes/Yes
Anti‐inflammatory treatment	2 (40)	No	Yes (mPRED)	Yes (mPRED)	No	No
Immunoglobulin treatment	5 (100)	First dose 2 g/kg	First dose and second dose 2 g/kg	First dose 2 g/kg	First dose 2 g/kg	First dose 2 g/kg
^13^N‐ammonia PET‐CT						
Global MFR (abnormal when < <2), gray zone 2–2.5 and normal >2.5	2 (40)	1.6	3.7	3.2	1.8	2.5
Echo parameters						
Normal echocardiogram at follow‐up	4 (80)	Normal	Normal	Normal	Abnormal^a^	Normal
LVDD *z*‐score	−0.13 (1.01)	0.81	−0.74	0.1	−1.57	0.74
LVSD *z*‐score	−0.66 (0.32)	−0.52	−0.83	−0.39	−0.43	−1.17
Septum *z*‐score	0.91 (0.38)	0.55	1.3	0.56	0.84	1.33
LVPW *z*‐score	0.62 (0.37)	0.21	1.00	0.31	0.58	1.00
LA *z*‐score	−0.72 (1.07)	0.68	0	−0.78	−1.82	–1.7
LVEF (%) (abnormal ≤55)	1 (20)	75	70	70	54	79
Doppler ultrasound of the brachial artery						
FMD (%)	6.38 (3.41)	9.36	–	3.92	10.07	2.14
FMD reference value (25th percentile [Hadi et al., 2005])	6.05 (0.40)	5.92	–	6.19	6.23	5.36
Endothelial dysfunction (i.e., below the FMD 25th percentile [Hadi et al., 2005])	2 (50)	No	–	Yes	No	Yes
Cardiopulmonary exercise test						
*V*O_2peak_ (ml/g/min)	26.3 (8.35)	22.5	17.4	28.6	–	36.8
Predicted *V*O_2peak_ (%) (<80% abnormal)	50.2 (12.8)	48.7	35.2	64.5	–	52.7
*V*O_2VAT_ (ml/g/min)	13.7 (4.64)	7.2	12.4	17.0	–	16.9
% from expected value	−54.0 (14.5)	−70.4	−60.8	−37.3		−47.6
*V*O_2VAT_ (%)/predicted *V*O_2_peak (<40 abnormal)	27.8 (9.73)	15.6	25.1	38.2	–	32.3
OUES (L/min)	1.20 (0.14)	1.2	1.0	1.3	–	1.3
OUES/kg	31.7 (10.1)	20.0	30.3	31.7	–	44.8
V_E_/*V*CO_2_ slope (units) (>31 abnormal)	34.8 (4.77)	41.7	31.8	34.4	–	31.4
% from expected value	12.6 (9.45)	25.6	3.0	10.0	–	12.0
PetCO_2_ rest (mmHg) (<35 abnormal)	29.7 (4.27)	24	33	33	–	29
% from expected value	−15.1 (12.0)	−31.4	−6.0	−6.0	–	−17.1
O_2_ pulse peak (ml/beat) (<14 abnormal)	6.25 (1.50)	7	4	7	–	7
% from expected value	−55.2 (10.5)	−50.0	−71.0	−50.0	–	−50.0
Laboratory data						
C‐reactive protein (0.3–10 mg/L)	1.29 (2.00)	<0.30	0.42	<0.30	4.85	0.57
D‐dimer (≤500 ng/ml)	623.5 (349 97592)	794	97.572	–	453	349
Fibrinogen (200–400 mg/dl)	289.2 (108.5)	311	190	217	465	263
Troponin (<0.004 ng/ml)	0.004 (0.001)	0.003	0.004	0.004	0.004	0.007

Abbreviations: BMI, body mass index; FMD, flow mediated dilatation; ICU, intensive care unit; LA, left atrium diastolic diameter; LVDD, left ventricle diastolic diameter; LVEF, left ventricle ejection fraction; LVPW, left ventricle posterior wall thickness; LVSD, left ventricle systolic diameter; MFR, myocardial flow reserve; MIS‐C, multisystem inflammatory syndrome in children; OUES, oxygen uptake efficiency slope; Septum, interventricular septum thickness; V_E_, pulmonary ventilation; *V*O_2peak_, peak oxygen consumption; *V*O_2VAT_, oxygen consumption at ventilatory anaerobic threshold.

^a^ Patient 4 exhibited discrete pericardial effusion at. Categorical data were reported as percentages and continuous data as mean±standard deviation (SD) or median (range).

### 2.2 ^13^N‐ammonia PET‐CT imaging protocol

^13^N‐ammonia was produced by means of an on‐site cyclotron installed at our institution (PETtrace^™^ 880; GE Healthcare), by ^16^O(p,α) 13N. In this procedure, ^13^N‐ammonia is synthesized directly in the target water (in‐target production) by adding ethanol 5 mmol as a free radical scavenger to prevent the formation of the oxo anions. The radiochemical purity was >99.9% within 60 min from the end of the bombardment. Subjects were maintained in a fasting state for at least 6 h before the study and were told not to consume methylxanthine‐containing foods or beverages (coffee, chocolates, soft drinks and tea) for at least 24 h before the PET scan.

For measurement of myocardial blood flow (MBF) at rest and at pharmacological stress (adenosine‐induced hyperemia), ^13^N‐ammonia was administered intravenously (0.286 mCi/kg) over a 10‐s period, the intravenous line was flushed with additional saline over a 10‐s interval and standardized imaging protocols were performed according to the American Society of Nuclear Cardiology guidelines (Vries et al., 2006). Stress imaging was performed identically, after adenosine infusion over 6 min (0.142 mg/min/kg). The myocardial perfusion radiopharmaceutical was injected about halfway into the adenosine infusion (at 3 min), when maximal vasodilatation and myocardial hyperemia were assumed to occur. MBF was expressed as ml/g/min.

**FIGURE 1:**
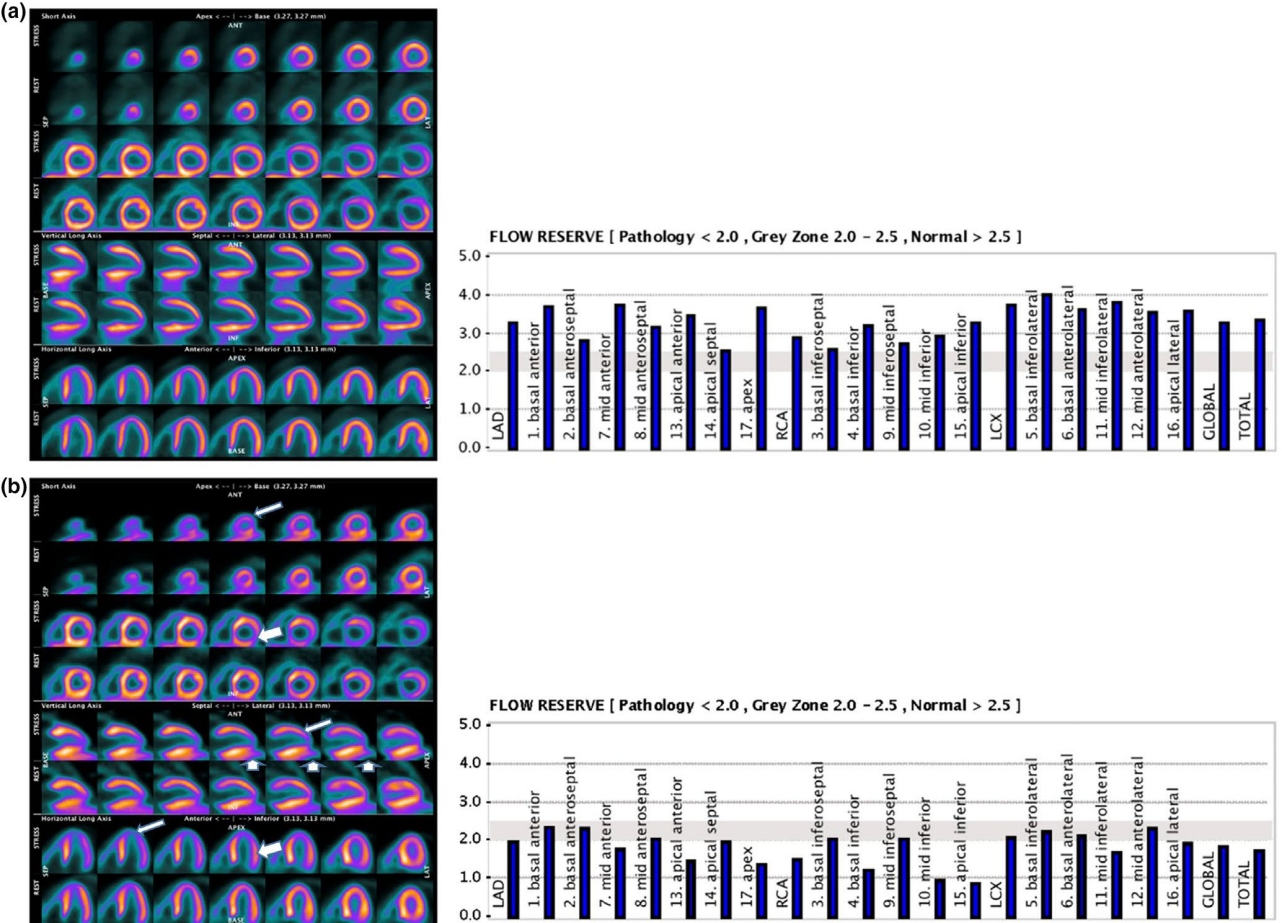
Illustrative data of ^13^N‐ammonia PET distribution: (a) Female patient, 9 years old, showed normal MFR. (b) Female patient, 8 years‐old, showed abnormal values for MFR. Transient perfusion defects in the anteroapical (narrow arrows), inferoapical (thick arrows) and inferolateral (upright arrows) territories during the stress phase were observed. MFR, myocardial flow reserve

**FIGURE 2:**
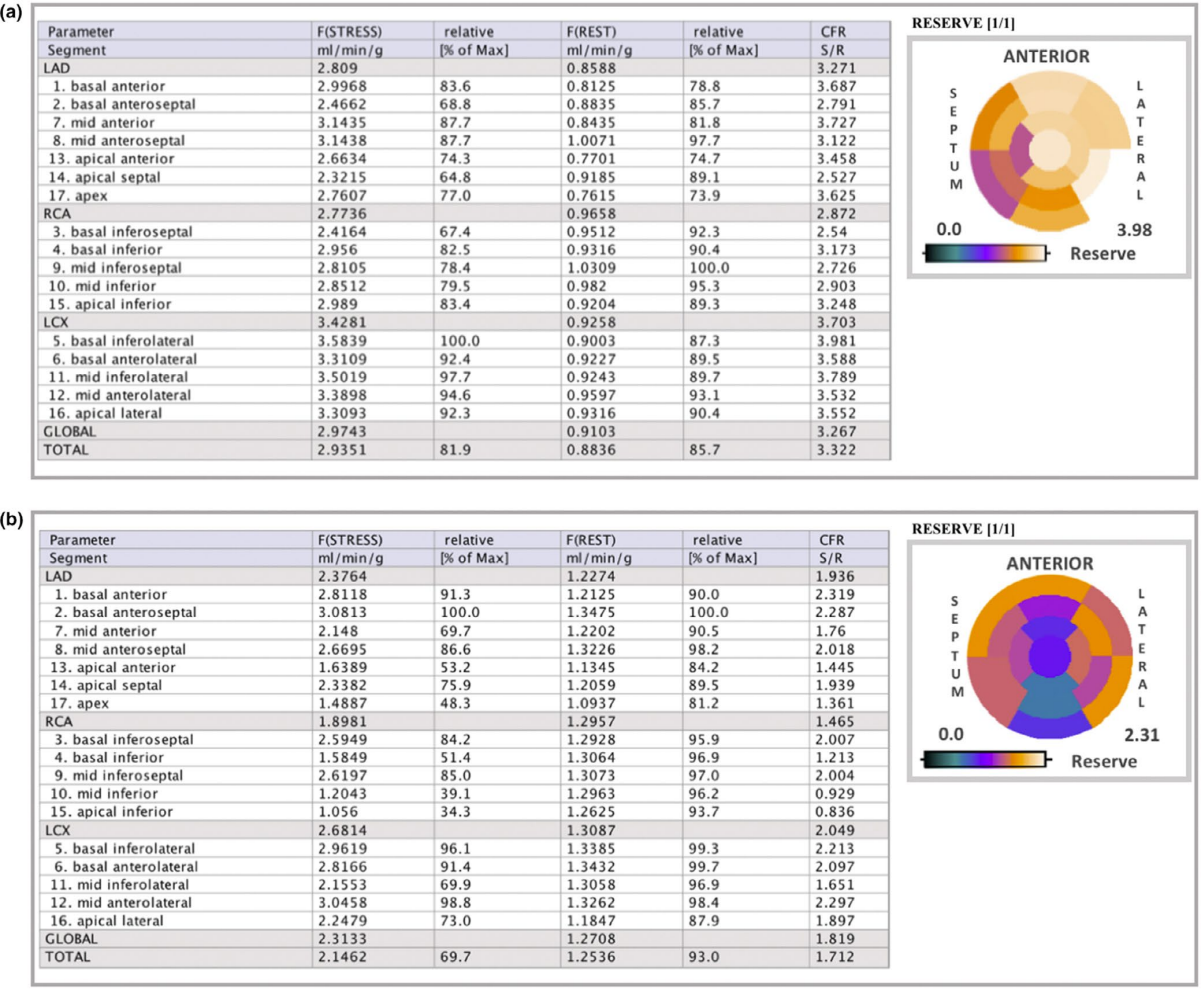
^13^N‐ammonia PET data. Polar maps of MBF values with the table on 17 American Heart Association (AHA) segments. (a) Female patient, 9 years old, showed normal values for MFR. (b) Female patient, 8 years old, showed abnormal values for MFR. In the bullseye illustrations, white‐to‐purple means normal values of MFR and blue‐to‐black means abnormal or decreased flow reserve. MBF, myocardial blood flow; MFR, myocardial flow reserve

The quantitative PET datasets were fused with CT using commercially available software (CardIQ Fusion, GE Healthcare). Quantitative MBF and myocardial flow reserve (MFR) was determined using the PMOD^™^ software package, version 3.4002 (PMOD Technologies LLC). Myocardial and blood‐pool time‐activity curves (TAC) were obtained from dynamic frames corrected for radioisotope decay. Segmental MBF was measured in each phase (rest and stress adenosine) by the model fitting of the blood pool and myocardial TACs, corrected for spill‐over and partial volume. MFR was calculated as the ratio of stress MBF over the rest MBF (the 17‐segment model according to the American Society of Nuclear Cardiology recommendations). For each left ventricle (LV) segment (see Figure 2), MFR at right coronary artery (RCA), left circumflex artery (LCX), left anterior descending (LAD) and MFR global were considered abnormal when <2, in gray zone when between 2 and 2.5 and normal when >2.5 (Duarte‐Neto et al., 2021) (see Figures 1 and 2 for illustrative exams).

### 2.3 Standard echocardiography

Standard transthoracic echocardiography was performed according to the recommendations of the American Society of Echocardiography (Farooqi et al., 2021). Cardiac chamber dimensions were obtained using two‐dimensional mode and left ventricle ejection fraction (LVEF) was calculated by Simpson's method (normal LV EF ≥55%) (Farooqi et al., 2021). The *z*‐score values of cardiac chambers were calculated according to Lopez et al (normal values between 2 and < +2.5). The equipment used was a Philips Affiniti 70, with multifrequency transducers (S 5‐1 and S 8‐3 MHz).

### 2.4 Brachial FMD

FMD was evaluated according to current guidelines (Feldstein et al., 2020) using a high‐resolution Doppler ultrasound machine (LOGIQ e PRO—GE Healthcare) equipped with a 4.0–12.0 MHz linear transducer. Initially, participants were positioned in the supine position with their right arm extended at an angle of ~80° from the torso. Longitudinal images of the brachial artery diameter were taken using the B‐mode ultrasound, and simultaneous pulse‐waved Doppler blood flow velocity was obtained using a 60° intonation angle with the sample volume placed in mid‐artery and aligned with the blood flow. Initially, a 1‐min baseline recording of the brachial artery diameter and blood flow velocity was performed. Then, the ischemic stimulus was performed by inflating a cuff placed in the forearm to 60 mmHg above the patient's resting systolic pressure for 5 min. Recordings were resumed 30 s before cuff deflation and continued for 3 min thereafter. Brachial artery diameter and shear rate (4 × mean blood velocity/internal diameter) were analyzed by a blinded evaluator using a semi‐automatic edge‐detection and wall‐tracking software (Cardiovascular Suite, Quipu^®^). FMD was calculated as the percentage change of the brachial artery diameter after cuff release in relation to baseline brachial artery diameter [FMD = (baseline diameter−peak diameter baseline diameter) × 100]. To describe the relevant shear rate stimulus for FMD, we also calculated the area‐under‐the‐curve of the shear rate up to the peak diameter (SRAUC). FMD lower than the age‐ and sex‐specific 25th percentile (Hadi et al., 2005) was considered as suggestive of endothelial dysfunction.

### 2.5 Cardiopulmonary exercise test

A symptom‐limited maximal cardiopulmonary exercise test was carried out on a treadmill (Centurion model 300; Micromed) using a ramp protocol test at a controlled room temperature (21–23°C). Peak oxygen consumption (*V*O_2peak_), oxygen consumption at ventilatory anaerobic threshold (*V*O_2VAT_), oxygen uptake efficiency slope (OUES), heart rate‐oxygen consumption relationship (HR/*V*O_2_ slope), oxygen pulse at peak of exercise (O_2_ pulse peak), V_E_/*V*CO_2_ slope were measured breath‐by‐breath through a computerized system (MetaLyzer 3B; Cortex). One patient (P4) was prohibited to perform the test by the cardiologist because she had lower % LVEF and persistent discrete pericardial effusion. Reference values from healthy children sorted by age and sex were used for identifying abnormal exercise capacity (Holder et al., 2021; Hossri et al., 2019).

## 3 RESULTS

The main findings can be seen in Table 1. P1 and P4 exhibited homogeneous rest but heterogeneous stress perfusion with perfusion defects developed in the slightly dilated left ventricular cavity, suggesting stress‐induced myocardial ischemia associated with MFR lower than 2.0 (LAD: 1.2; RCA: 2.0; and LCX: 2.1 and LAD: 1.9; RCA: 1.4; and LCX: 2.0) respectively, see P4 in Figures 1b and 2b.

All patients showed signs suggesting normal coronary arteries (all score‐*z* ≥2.5) (Farooqi et al., 2021) by standard echocardiogram at post‐discharge, except for one patient (P4).

FMD assessment was not completed in one participant (P2) who experienced significant discomfort during the procedure. Of the remaining four participants, mean (SD) FMD% was 6.38 ± 3.41. Participants P1 and P4 presented with preserved FMD, while participants P3 and P5 had reduced FMD suggestive of endothelial dysfunction.

Mean (SD) *V*O_2peak_ was 26.3 ± 8.4 ml/kg/min. All patients showed abnormal *V*O_2peak_, with lower predicted values (range: 35.2–64.5%). Similarly, all patients had lower predicted values for *V*O_2VAT_ (range: 15.6–38.2%), OUES (range: 1.0–1.3 L/min) and O_2_ Pulse (range: 4–7 ml/beat). A ventilatory inefficiency was also identified, considering the mean (SD) value of V_E_/*V*CO_2_ Slope 34.8 ± 4.8 units (Hossri et al., 2019). Collectively, these findings indicate an impairment in cardiorespiratory and oxidative metabolism during physical exercise.

P1, P2, and P4 had abnormal values for D‐dimer and fibrinogen, respectively. The other parameters were within normal range.

## 4 DISCUSSION

This study reveals novel pathological findings in MIS‐C patients which may help optimize treatment protocols in this condition. P1 and P4 exhibited impaired MFR, whereas P3 and P5 showed reduced endothelial function. All patients showed dysfunctional cardiorespiratory responses to a maximal exercise test.

To our knowledge, this is the first study to investigate myocardial perfusion and blood flow by PET imaging in a case series of MIS‐C. This robust technique has been considered useful in clinical decision‐making for patients with suspected coronary artery disease, as it can detect multivessel ischemia that could otherwise appears as normal on stress imaging if ischemia is global and balanced among all coronary territories (Lopez et al., 2010). The ratio of MBF at stress over rest is labeled MFR. It is primarily controlled by the release of local metabolites such as adenosine or nitric oxide. As the heart has minimal ability to increase oxygen extraction and rely on anaerobic metabolism, increased metabolic demands of the heart are met primarily via increases in coronary blood flow. In the absence of obstructive epicardial coronary artery disease, as it was the case of our patients presumably, coronary blood flow is primarily controlled by changes in resistance in the small arteries and arterioles (i.e., microvasculature), which play an important role in myocardial perfusion in general in regional and transmural distribution. Herein two patients showed abnormal MFR, which could be a consequence of coronary microvascular dysfunction, resulting from vasomotor dysregulation or endothelial dysfunction of the small coronary arterioles. In fact, our data add to post‐mortem evidence suggesting that coronary microvascular involvement appears to comprise COVID‐19/MIS‐C pathophysiology (Penner et al., 2021). Of relevance, we also observed brachial endothelial dysfunction (as assessed by FMD) in two other patients different from those with abnormal findings upon PET imaging, suggesting that vascular involvement is not restricted to microvasculature in MIS‐C. Collectively, the present results may be of clinical relevance since vascular dysfunction is a potentially reversible condition that is associated with future cardiovascular events (Singh et al., 2003).

Another striking finding was the abnormal cardiorespiratory response during exercise. Some metrics of impaired oxidative metabolism (e.g., lower *V*O_2VAT_ and OUES) and ventilatory inefficiency (e.g., higher VE/*V*CO_2_ slope) were below normal values for all patients. Also, all patients showed lower *V*O_2peak,_ which is an independent risk factor associated with poor prognosis in several diseases and all‐cause mortality in general population (Thijssen et al., 2019). Rehabilitating cardiopulmonary capacity emerges as a potential therapeutic goal in MIS‐C to prevent any cardiac events, improve patients’ fitness and restore performance in daily living activities.

This study has limitations. First, given the paucity of ^13^N‐ammonia PET/CT studies and a large normal database in children, the arbitrary threshold limit (i.e., 2.5 ml/g/min) used to separate normal from abnormal MBF has not been yet validated in the pediatric population. Second, the low number of patients enrolled, and the lack of a control group without MIS‐C and the longitudinal assessments preclude any causative inferences and insights on natural course of the syndrome. Therefore, studies assessing the frequency, predictors, clinical repercussion, and mechanisms of the cardiovascular and pulmonary findings described herein are warranted.

In conclusion, we reported on novel pathophysiological findings in MIS‐C patients (i.e., reduced myocardial perfusion, cardiopulmonary capacity, and endothelial function), which advances the knowledge on this newly described condition and may help tailor better treatments for these patients. In‐depth investigation using ^13^N‐ammonia PET‐CT imaging, brachial FMD, and cardiopulmonary exercise testing provides supplementary information that might be helpful in clinical decision‐making in MIS‐C care.

## CONFLICT OF INTEREST

We declare no competing interests.

## AUTHOR CONTRIBUTIONS

All the authors contributed substantially to the conception and design of the study and in the analysis and interpretation of data. All authors revised the work critically and approved the final version.

## ETHICS APPROVAL STATEMENT AND CLINICAL TRIAL REGISTRATION

This study was approved local ethics committee (protocol #37460620.8.0000.0068) and registered at ClinicalTrials.gov (NCT04659486).

## Data Availability

Access to de‐identified data or related documents can be requested through the submission of a proposal with a valuable research question, necessary data protection plan, and ethical approvals. A contract will be signed. Data requests should be addressed to the corresponding author.
